# Age is not a determinant factor in susceptibility of broilers to H5N2 clade 2.3.4.4 high pathogenicity avian influenza virus

**DOI:** 10.1186/s13567-016-0401-6

**Published:** 2016-11-21

**Authors:** Kateri Bertran, Dong-Hun Lee, Charles Balzli, Mary J. Pantin-Jackwood, Erica Spackman, David E. Swayne

**Affiliations:** Exotic and Emerging Avian Viral Diseases Research Unit, Southeast Poultry Research Laboratory, US National Poultry Research Center, Agricultural Research Service, US Department of Agriculture, 934 College Station Rd, Athens, GA 30605 USA

## Abstract

In 2014–2015, the US experienced an unprecedented outbreak of H5 clade 2.3.4.4 highly pathogenic avian influenza (HPAI) virus. The H5N2 HPAI virus outbreak in the Midwest in 2015 affected commercial turkey and layer farms, but not broiler farms. To assess any potential genetic resistance of broilers and/or age-related effects, we investigated the pathogenesis and transmission of A/turkey/Minnesota/12582/2015 (H5N2) (Tk/MN/15) virus in commercial 5-week-old broilers, 8-week-old broilers, and >30-week-old broiler breeders. The mean bird lethal dose (BLD_50_) was 5.0 log_10_ mean egg infectious dose (EID_50_) for all age groups. The mean death time (MDT) was statistically not different among the three age groups, ranging between 3.2 and 4.8 days. All broilers that became infected shed high levels of virus with transmission to contacts and demonstrated severe pathology. Mortality and virus shedding results indicated that age is not a determinant factor in susceptibility of broilers to H5N2 clade 2.3.4.4 HPAI virus. Previously, the Tk/MN/15 virus had a BLD_50_ of 3.6 log_10_ EID_50_ and MDT of 2 days in White Leghorn chickens and a BLD_50_ of 5.0 log_10_ EID_50_ and MDT of 5.9 days in turkeys, suggesting that the broiler breed is less susceptible to Midwestern H5N2 virus than the layer breed but similarly susceptible to turkeys. Therefore, genetic resistance of broilers to infection may have accounted only partially for the lack of affected broiler farms in the Midwestern outbreaks, with other contributing factors such as fewer outside to on farm exposure to contacts, type of production management system or enhanced biosecurity.

## Introduction

The Asian-origin H5N1 A/goose/Guangdong/1/1996 (Gs/GD) lineage of high pathogenicity avian influenza (HPAI) virus has become widespread across several continents, affecting wild birds, poultry, and humans. In late 2014, Eurasian H5N8 and reassortants H5N2 and H5N1 Gs/GD lineage clade 2.3.4.4 HPAI viruses were reported in North America [1]. The initial incursion of this viral lineage into the US was detected in December 2014 in Washington state in a wild Northern pintail duck (*Anas acuta*) [2]. The H5N2 virus was a reassortant containing five Eurasian avian influenza (AI) virus gene segments [including the H5 2.3.4.4 hemagglutinin (HA)] and three North American wild bird lineage low pathogenic (LP) AI virus gene segments [2–4]. Over the next 7 months, the US experienced the worst HPAI event for US poultry producers: more than 7.5 million turkeys and 42.1 million chickens died or were culled during the control program [5] and imports of US poultry and poultry products from many different countries were banned [6].

While the majority of initial H5 HPAI viruses were detected in wild waterfowl, wild and captive birds of prey, and backyard flocks along the Pacific flyway [5, 7], most cases in the Midwest in 2015 affected commercial turkey and chicken layer premises [5]. Experimentally, H5N2 HPAI viruses isolated from the Midwestern poultry cases in 2015 were generally better adapted to White Leghorn chickens [8] and turkeys [9], as opposed to the index virus A/Northern pintail/Washington/40964/2014 (H5N2) which was more waterfowl adapted [10, 11] (adaptation meaning efficiency of replication and release of the virus from a specific host species, also associated to transmissibility [12]). Overall, the H5N2 virus likely adapted to chicken layers and turkeys during the early Midwestern outbreaks increasing infectivity and transmission, and facilitating spread [10]. Other epidemiological factors such as weather, husbandry conditions, flock density and composition, but especially breakdowns in farm biosecurity, may have also contributed to the increasing virulence and transmission in the Midwestern commercial farms.

Albeit HPAI viruses produce high morbidity and mortality in gallinaceous domestic poultry regardless of age [13–15], age-related susceptibility, in particular the severity of the clinical signs and the capacity of recovery, has been reported in turkeys and ostriches infected with LPAI viruses [16, 17]. In a natural outbreak of H5N1 influenza virus in commercial ducks in South Korea, 14-day-old meat ducks experienced increased morbidity and up to 12% mortality rate, with systemic microscopic lesions. In contrast, adult ducks at the affected breeder duck farms showed decreased egg production and feed consumption, but no mortality [18]. Experimentally, an effect of virus strain and host age on the resulting pathogenicity was observed in 2- and 5-week-old Pekin ducks infected with different Asian-origin HPAI H5N1 viruses [19]. Reproductively active adult Japanese quail (*Coturnix c. japonica*) had stronger immune responses than pubescent and aged birds, suggesting an age-related difference in immune function [20].

The different genetic background between layer-type and broiler-type chickens may have an effect not only on performance but also on genetic expression and immunological responses [21–23]. Differences in susceptibility between layers and broilers have been reported for AI virus infection [24–31]. Experimentally, the intravenous inoculation of high dose of A/chicken/Alabama/7395/75 (H4N8) caused more severe renal disease in White Leghorns than in broilers [24]. In another study, broiler breeds were identified as generally more resistant to A/chicken/Italy/13474/99 (H7N1) HPAI virus than layer breeds [29]. In an experimental study with low dose intranasal challenge with a reverse-genetics-derived rg-A/chicken/Indonesia/7/2003 (H5N1) HPAI virus, there were differences in mean death time (MDT) and mortality rates in congenic White Leghorn chickens, but these differences were mildly influenced by the major histocompatibility complex (MHC) gene and more so by non-MHC background genes [30]. Overall, these data suggest that variations in response to AI virus infection among breeds may be due to genetic-based natural resistance [25, 27–29].

During the outbreaks in the Midwest in 2015, turkey and layer-type chicken farms were affected but broiler farms were not [5], despite close proximity of the three types of production systems [32]. The absence of affected broiler farms could be the result of genetic resistance to infection and/or failure to introduce HPAI virus onto the farms, due to either lack of exposure or good biosecurity. Knowing species-, breed-, and age-related differences in susceptibility could decisively impact optimal management of outbreak control strategies. In the present study, pathogenesis and transmission dynamics of Midwestern H5N2 clade 2.3.4.4 HPAI virus were investigated in commercial broilers of three different ages in order to test the ability of the virus to produce infection in broilers, as opposed to layers, and assess age-related differences in susceptibility.

## Materials and methods

### Virus

The influenza A isolate A/turkey/Minnesota/12582/2015 (H5N2) (Tk/MN/15) was used as challenge virus. The virus was isolated from a turkey farm in Minnesota from a sample collected April 18^th^ 2015. The virus was propagated and titrated by allantoic sac inoculation of 9–10 day-old embryonating chicken eggs (ECE) by standard methods [33].

### Phylogenetic analysis

The Tk/MN/15 virus was selected because it is representative of the Midwest H5N2 cluster both phenotypically [8] and by phylogenetic analysis. For the phylogenetic analysis, a total of 82 HA gene segments were used in this study. Specifically, the nucleotide sequences of 81 H5 HPAI viruses identified from December 2014 to June 2015 in the US were analyzed together with the HA segment of Korean H5N8 virus identified in January 2014 that were available in the GenBank. The nucleotide sequences of HA segment were aligned using MUSCLE [34]. The Bayesian relaxed clock phylogenetic analyses were done using BEAST v1.8.2 [35]. We applied an uncorrelated lognormal distribution relaxed clock method, the Hasegawa-Kishino-Yano nucleotide substitution model and the Bayesian skyline coalescent prior. A Markov Chain Monte Carlo method to sample trees and evolutionary parameters was run for 5.0 × 10^7^ generations. At least three independent chains were combined to ensure adequate sampling of the posterior distribution of trees. BEAST output was analyzed with TRACER v1.4 with 10% burn-in. The FigTree v.1.4.2 program was used to construct and visualize the maximum clade credibility tree.

### Animals and housing

Crossed-line WPR × Cornish broiler birds from a commercial producer in GA, USA (an AI-free state during the outbreak) were utilized. The birds had been reared under field management conditions, including a typical US vaccination program which comprised a broad range of vaccines, but not exposed to or vaccinated against AI virus. One week prior to challenge, birds were moved from the field to the controlled environment in SEPRL ABSL-3 facilities to maintain biosafety and biosecurity barriers for H5N2 HPAI virus challenge. Birds were obtained at three different ages: 5-week-old (5w) broilers as early processing age, 8-week-old (8w) broilers as late processing age, and >30-week-old broiler breeders (>30w). Broiler breeders were in lay; all eggs produced during the study were discarded. Prior to inoculation, 30% of chickens of each age group were ensured to be serologically negative for AI virus infection as determined by hemagglutinin inhibition (HI) test. Also, oral swabs were collected before challenge to confirm absence of virus shedding as determined by quantitative real-time RT-PCR (qRRT-PCR). Each experimental group was housed separately in negative pressure isolators with HEPA-filtered ventilation within the animal biosafety level 3 enhanced facilities at Southeast Poultry Research Laboratory. The birds had ad libitum access to feed and water.

### Experimental design and sampling

#### Infectivity and transmission

To evaluate the mean bird infectious (BID_50_) and lethal (BLD_50_) doses at different ages, each age group was divided into three groups (*n* = 5/group), each inoculated intranasally with 2 (low dose), 4 (medium dose), or 6 (high dose) log_10_ mean egg infectious dose (EID_50_)/0.1 mL of Tk/MN/15 virus (Table 1). Sham challenged birds of each age were inoculated intranasally with 0.1 mL of sterile allantoic fluid diluted 1:300 in brain heart infusion (BHI) media (Becton, Dickinson and Company, Sparks, MD, USA). The inoculum titers were subsequently verified by back titration in ECE as 1.9–2.1 (low dose), 3.9–4.5 (medium dose), and 5.7–6.3 (high dose) log_10_ EID_50_/0.1 mL. To evaluate the transmissibility of each isolate, three non-inoculated hatch-mates were added to each dose group at 1 day post-challenge (dpc). Clinical signs were monitored daily. Oral swabs were collected from all birds on 2 and 4 dpc, placed in 1.5 mL of BHI with penicillin (2000 units/mL; Sigma Aldrich), gentamicin (200 μg/mL; Sigma Aldrich) and amphotericin B (5 μg/mL; Sigma Aldrich), and stored at −80 °C until use. Severely sick birds were euthanized and counted as dead for the next day in MDT calculations. At 14 dpc, surviving birds were bled to evaluate antibody titers and euthanized by intravenous administration of sodium pentobarbital (100 mg/kg body weight).

**Table 1:** **Transmission study design with mortality results and mean bird infectious and lethal doses**

Bird type (age)	Dose (log_10_ EID_50_)	Mortality (MDT expressed as dpc or dpe)^a^		BID_50_ and BLD_50_ (log_10_)
		Inoculated	Contact	
Broilers (5w)	2	0/5	0/3	5.0
	4	0/5	0/3	
	6	5/5 (4.8)	3/3 (5.3)	
Broilers (8w)	2	0/5	0/3	5.0
	4	0/5	0/3	
	6	5/5 (3.2)	3/3 (4.3)	
Broiler breeders (>30w)	2	0/5	0/3	5.0
	4	0/5	0/3	
	6	5/5 (3.2)	3/3 (5.7)	
	Sham inoculated	0/2	0/2	na
SPF White Leghorns (4w)^b^	2	0/5	0/3	3.6
	4	3/5 (2.3)	0/3	
	6	8/8 (2)	2/2 (4)	
Commercial broad breasted white turkeys (4w)^c^	2	0/5	0/3	5.0
	4	0/5	0/3	
	6	21/21 (5.9)	3/3 (8)	

5w: 5-week old broilers, 8w: 8-week old broilers, >30w: >30-week-old broiler breeders, BID_50_: mean bird infectious dose, BLD_50_: mean bird lethal dose, dpc: days post-challenge, dpe: days post-exposure, EID_50_: mean egg infectious dose, MDT: mean death time, na: not applicable.

^a^#dead birds × dpc/total dead birds.

^b^For comparative purposes, data from DeJesus et al. [8] on 4-week old SPF White Leghorn chickens challenged with Tk/MN/15 virus have been added.

^c^For comparative purposes, data from Spackman et al. [9] on 4-week old commercial broad breasted white turkeys challenged with Tk/MN/15 virus have been added.

#### Pathogenesis

For each age group, two additional birds were challenged with the high dose (5.7–6.3 log_10_ EID_50_/0.1 mL) of Tk/MN/15 virus to harvest tissue. Upon death, the birds were necropsied and portions of nasal cavity, brain, thymus, trachea, lung, proventriculum, duodenum, pancreas, jejunum–ileum, spleen, kidney, adrenal and gonad, liver, skeletal muscle, comb, and heart were collected in 10% buffered formalin (Thermo Fisher Scientific, Waltham, MA, USA) for histopathologic evaluation and for detection of AI virus in tissues by immunohistochemistry. Brain, spleen, heart, and lung were also collected in BHI with antibiotics to a 1% (wt/vol) concentration for viral RNA quantification by qRRT-PCR.

### Viral RNA quantification in swabs and tissues

Swabs and tissues in BHI were processed for qRRT-PCR to determine viral RNA titers. Viral RNA was extracted using MagMAX™-96 AI/ND Viral RNA Isolation Kit^®^ (Ambion, Inc.) following the manufacturer’s instructions. In tissue homogenates, and in order to standardize the amount of nonspecific RNA from the tissue, the resulting viral RNA extracts were quantified by NanoDrop™ 1000 Spectrophotometer (Thermo Fisher Scientific) following the manufacturer’s instructions and accordingly diluted with phosphate buffered saline to obtain 50 ng/μL. The resulting viral RNA extracts, diluted (tissue homogenates) or undiluted (swabs), were quantified by one-step qRRT-PCR which targets the influenza matrix gene [36] using 7500 FAST Real-time PCR System (Applied Biosystems, Foster City, CA, USA). The standard curves for viral RNA quantification were established with RNA extracted from dilutions of the same titrated stocks of the challenge viruses. The limit of detection for Tk/MN/15 virus was 2.0 log_10_ EID_50_/mL (3.0 log_10_ EID_50_/g for tissue homogenates); for statistical purposes, qRRT-PCR negative samples were given a numeric value of 1.9 log_10_ EID_50_/mL (2.9 log_10_ EID_50_/g).

### Statistical analysis

The D’Agostino and Pearson test was used to assess the normality of distribution of investigated parameters. All parameters in our study were not normally distributed. Significant difference for mean viral titers in tissues between groups was analyzed using Kruskal–Wallis test or Mann–Whitney test (GraphPad Prism™ Version 5 software). A *p* value of <0.05 was considered to be significant.

### Histopathology and immunohistochemistry

Tissues in 10% formalin were processed for routine hematoxylin/eosin staining. Tissues were also processed for immunohistochemical staining using a mouse-derived monoclonal antibody (P13C11, developed at SEPRL) specific for type A influenza virus nucleoprotein, as previously described [10, 37].

### Serology

Sera samples were tested by HI assays against the challenge antigen. The antigen was prepared as previously described [38] and the HI assays were performed according to standard procedures [39]. Titers were calculated as the reciprocal of the last HI positive serum dilution and samples with HI titers below 8 (2^3^) were considered negative.

## Results

### Phylogenetic analysis

**Figure 1:**
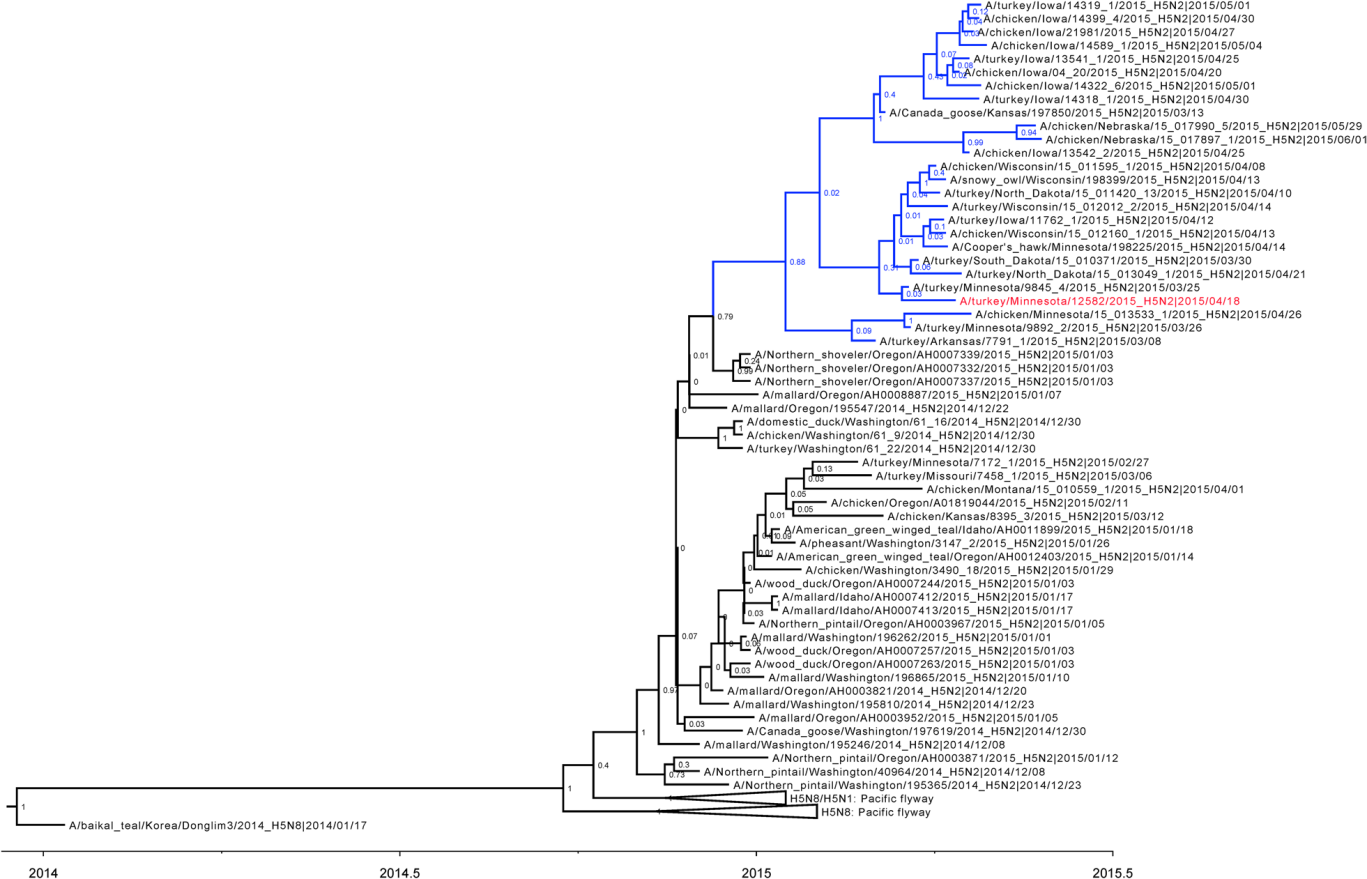
**Relaxed clock molecular phylogenetic tree for H5 clade 2.3.4.4 HPAI viruses in the US.** At each node, the number indicates a posterior probability. Blue branches represent the Midwest H5N2 cluster. The virus used in this study, A/turkey/Minnesota/12582/2015 (H5N2), is colored in red.

The HA gene of H5N1, H5N2, and H5N8 viruses were grouped by subtypes and geographical regions (Figure 1). The H5N2 HPAI viruses that affected commercial turkey and layer farms in the Midwestern region in 2015 clustered together in the genetically distinct cluster “Midwest H5N2”. Each gene of the isolates within the Midwest H5N2 cluster shared high sequence homology at nucleotide level (PB2: 99.6–100%, PB1: 99.3–100%, PA: 99.4–100%, HA: 99.3–100%, NP: 99.4–100%, NA: 99.5–100%, M: 99.6–100%, and NS: 99.4–100%). The phylogenetic analysis confirmed that the Tk/MN/15 virus used in this study belongs to this Midwest H5N2 cluster.

### Infectivity and transmission

The BID_50_ and BLD_50_ were determined for each of the three ages. Birds were considered infected if they had detectable virus along with clinical disease and mortality, or if they seroconverted by 14 dpc. For all age groups, none of the chickens inoculated with the low and medium doses died (Table 1). However, 100% of the chickens inoculated with the high dose of Tk/MN/15 virus became infected and died with a MDT of 4.8 dpc for 5w broilers and 3.2 dpc for both 8w broilers and >30w breeders (Table 1); MDTs were not significantly different among group ages (*p* > 0.05). The surviving inoculated birds did not show evidence of clinical disease and they were all serologically negative based on HI data, thus all survivors were considered uninfected. The BID_50_ and the BLD_50_ for Tk/MN/15 virus were the same in this study and resulted in 5.0 log_10_ BLD_50_ for all the age groups (Table 1).

**Figure 2:**
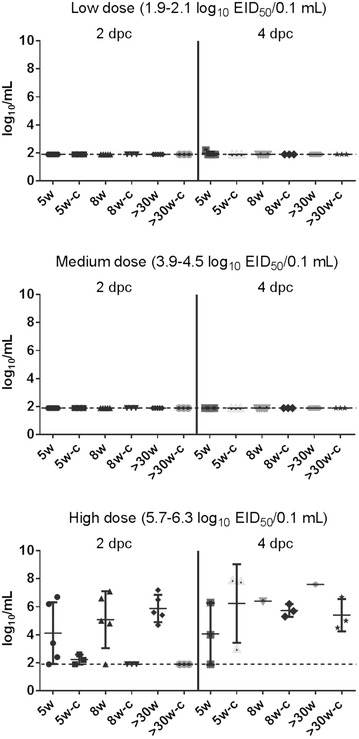
**Mean viral oral shed from broilers directly inoculated or contact exposed with A/turkey/Minnesota/12582/2015 (H5N2) virus.** Virus detection by qRRT-PCR at 2 and 4 dpc (or 1 and 3 dpe). The limit of detection for Tk/MN/15 virus was 2.0 log_10_ EID_50_/mL; therefore, qRRT-PCR negative samples were given a numeric value of 1.9 log_10_ EID_50_/mL. Intranasally inoculated 5-week old broilers (5w), 8-week old broilers (8w), and >30-week-old broiler breeders (>30w); contact exposed 5-week old broilers (5w-c), 8-week old broilers (8w-c), and >30-week-old broiler breeders (>30w-c).

Quantitation of viral shedding was performed by qRRT-PCR using extrapolation of a standard curve generated with the challenge virus via virus isolation and titration. Birds inoculated with the low and medium doses of Tk/MN/15 virus did not shed detectable levels of virus (Figure 2). In contrast, birds in all three age groups inoculated with the high dose of Tk/MN/15 virus shed high quantities of virus at 2 and 4 dpc before dying (Figure 2). At 2 dpc, mean virus titers of 4.1, 5.1, and 5.9 log_10_ EID_50_/mL were shed orally by 5w, 8w and >30w, respectively (Figure 2). Virus titers shed orally at 2 dpc were statistically not different among the three age groups (Kruskal–Wallis test, *p* > 0.5). At 4 dpc, mean virus titers of 4.0, 6.4, and 7.6 log_10_ EID_50_/mL were shed orally by 5w, 8w and >30w, respectively (Figure 2). Statistical analysis could not be performed for 4 dpc virus shedding due to the low number of birds alive for sampling.

The bird transmission studies were conducted by introducing three naïve birds of the same age into the isolators 24 h after the direct intranasal inoculation of five chickens of the corresponding age. Similar to what was observed for the inoculated birds, none of the contact chickens placed at the low and medium doses died (Table 1). However, 100% of the contact chickens placed at the high dose of Tk/MN/15 virus became infected and died with a MDT of 5.3, 4.3, and 5.7 days post-exposure (dpe) for 5w, 8w and >30w, respectively (Table 1). Virus was not detected in the oral swabs of contact exposed birds placed at the low and medium challenge doses of any of the three age groups (Figure 2). This, together with the lack of clinical disease, mortality, or seroconversion (data not shown) indicates that surviving contact exposure birds from low and medium challenge doses did not become infected. In contrast, contact exposure birds placed at the high dose of all three age groups shed high quantities of virus at 4 dpc (3 dpe) before dying, ranging from 5.4 to 6.2 log_10_ EID_50_/mL; two of three 5w-contact broilers were already shedding virus (2.2 and 2.6 log_10_ EID_50_/mL) at 1 dpe (2 dpc in the figure) (Figure 2).

### Pathogenesis

**Figure 3:**
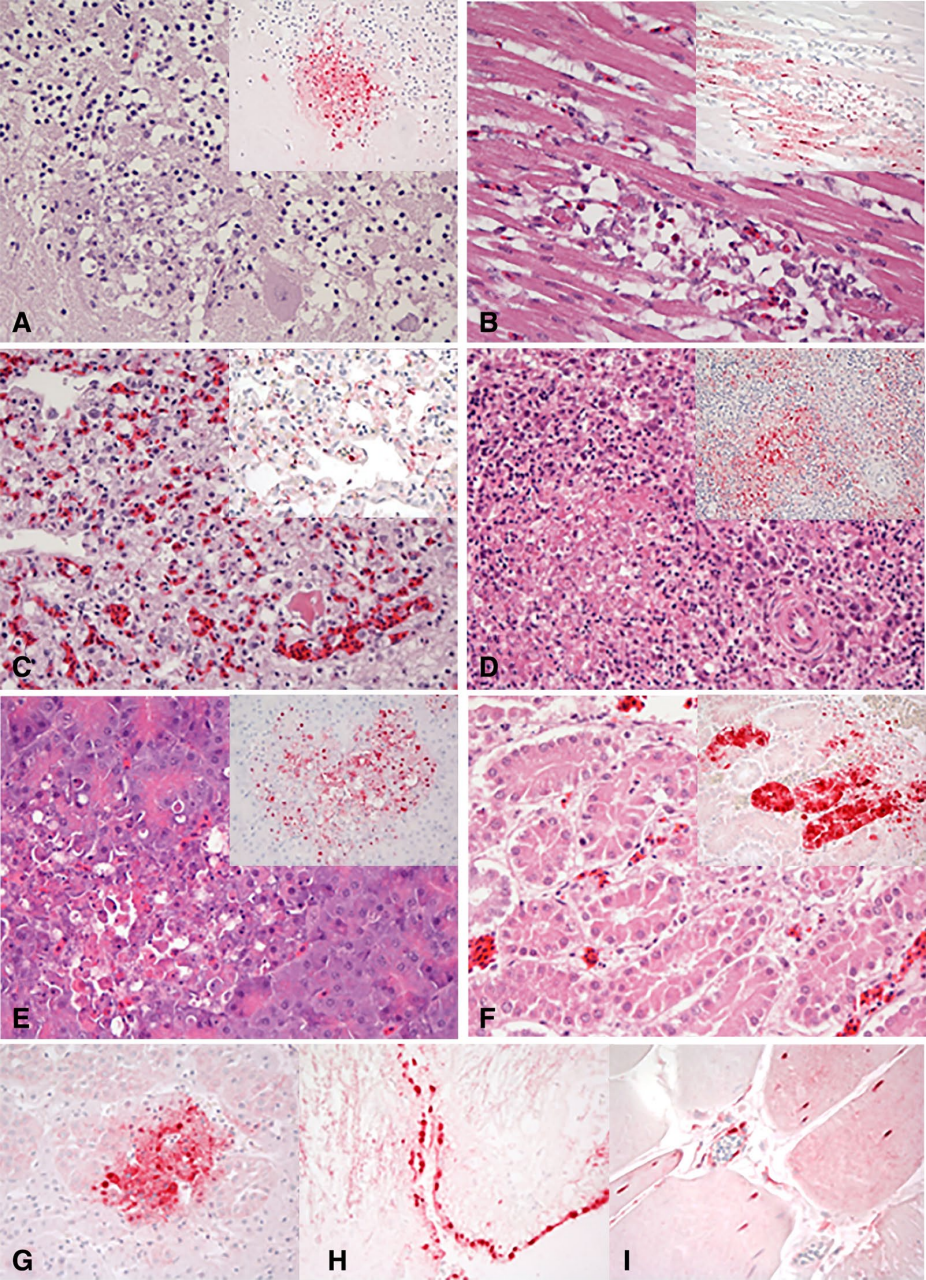
**Histopathological findings in broilers intranasally inoculated with high dose of A/turkey/Minnesota/12582/2015 (H5N2) virus.** 5-week old broilers (5w), 8-week old broilers (8w), >30-week-old broiler breeders (>30w); ×40; immunohistochemical detection of viral antigen staining in red. **A** Cerebellum, 5w. Vacuolation of the molecular and granular layers of the cerebellum with necrosis of the Purkinje neurons. Viral antigen present in the neuropil of both layers (inset). **B** Heart, 8w. Degeneration and necrosis of myocardiocytes. Extensive intranuclear and intracytoplasmic viral antigen of myocardiocytes (inset). **C** Lung, 5w. Severe congestion, interstitial edema, and interstitial heterophilic and monocytic infiltration. Viral antigen in epithelium of air capillaries and vascular endothelium (inset). **D** Spleen, 5w. Multifocal areas of necrosis and depletion of the white pulp. Viral antigen in mononuclear cells (inset). **E** Pancreas, >30w. Multifocal areas of degeneration of pancreatic acinar cells. Viral antigen in acinar cells (inset). **F** Kidney, 8w. Focal necrosis of tubular epithelium. Extensive intranuclear and intracytoplasmic viral antigen of tubular epithelial cells (inset). **G** Adrenal gland, 8w. Diffuse intranuclear and cytoplasmatic viral antigen in corticotropic cells. **H** Brain, >30w. Viral antigen in ependymal cells of the ventricles. **I** Skeletal muscle, 8w. Intranuclear and intracytoplasmic viral antigen of myocytes and vascular endothelium.

Two birds from each age group were challenged with the high dose of Tk/MN/15 virus to serve as a source of tissues. Mild illness was evident by 2 dpc in all age groups, consisting of nonspecific clinical signs such as ruffled feathers, listlessness, and infraorbital swelling. Necropsies were performed at 3 dpc on two dead birds per age group. Gross lesions were consistent in the three age groups, and included multifocal necrosis in the pancreas sometimes accompanied by hemorrhagic duodenum, splenomegaly and renomegaly with parenchymal mottling, and petechial hemorrhages on the pericardial fat. The two necropsied >30w breeders also had hemorrhagic tracheas. Similar type and severity of histological lesions, as well as viral antigen detection by immunohistochemistry, were observed for the three age groups (Figure 3). Multifocal necrosis with viral antigen was widespread in the parenchymal cells of most tissues, especially prominent in brain, heart, lung, spleen, pancreas, kidney, adrenal gland, and ovaries (in >30w broiler breeders) (Table 2; Figure 3). Viral antigen staining was frequently observed in capillary endothelial cells of various tissues but not as widespread as observed with H5N1 Gs/GD lineage viruses [14, 37, 40].

**Table 2:** **Microscopic lesions and distribution of AI nucleoprotein antigen in tissues by immunohistochemistry**

Tissue^a^	Commercial broiler			SPF layer	Viral antigen stained cell types	Type of lesion
	5w	8w	>30w	4w^b^		
Trachea	+	+	−	++	Pseudostratified epithelial cells	Focal necrosis with mild lymphoplasmacytic inflammatory infiltrate
Lung	+++	+++	+	+++	Epithelium of air capillaries, mononuclear cells, endothelial cells	Moderate congestion, necrosis, and monocyte inflammatory infiltrate
Duodenum	+	−	−	na	Macrophages	NSL
Cecal tonsils	+	−	−	+	Epithelial cells of villus, cells of the muscularis externa, macrophages	Focal necrosis with lymphoplasmacytic inflammatory infiltrate
Pancreas	+++	++	+++	++	Acinar cells	Mild degeneration of individual pancreatic acinar cells
Liver	+	+	+	+	Kupffer cells, hepatocytes, macrophages	Focal necrosis with lymphoplasmacytic inflammatory infiltrate, perivascular cuffing
Kidney	++	+++	++	+	Tubular epithelial cells	Focal necrosis of tubular epithelium with lymphoplasmacytic inflammatory
Adrenal gland	++	+++	++	++	Corticotropic cells	Multifocal areas of necrosis with mononuclear inflammatory infiltrate
Spleen	+++	++	+++	++	Mononuclear cells	Multifocal areas of necrosis, hemorrhages, depletion white pulp
Thymus	+	+	+	++	Mononuclear cells	Focal necrosis, mild lymphocyte depletion, apoptotic lymphocytes
Heart	+++	+++	+++	+++	Myocardiocytes	Multifocal necrosis of myocardiocytes
Skeletal muscle	+	++	+	na	Myocytes, connective tissue, endothelial cells	NSL
Brain	+++	+++	+++	+++	Neurons, Purkinje cells, ependymal cells, glial cells	Malacia in cortex, necrosis of ependymal cells of ventricles and epithelial cells of choroid plexus, chromatolysis of Purkinje cells, lymphoplasmacytic infiltrate
Ovaries	na	na	+++	na	Tegument/interstitial tissue, granulocytes	Focal necrosis with lymphoplasmacytic inflammatory infiltrate
Infundibulum	na	na	+	na	Ciliated epithelial cells	NSL

4w: 4-week old SPF White Leghorn layers, 5w: 5-week old broilers, 8w: 8-week old broilers, na: not applicable, NSL: no significant lesions.

^a^Tissues not present appeared overtly normal on histopathological analysis and did not show positive immunohistochemical staining.

^b^For comparative purposes, data from DeJesus et al. [8] on 4-week old SPF White Leghorn chickens challenged with Tk/MN/15 virus have been added.

− = no positive cells; + = single positive cells; ++ = scattered groups of positive cells; +++ = widespread positivity.

**Figure 4:**
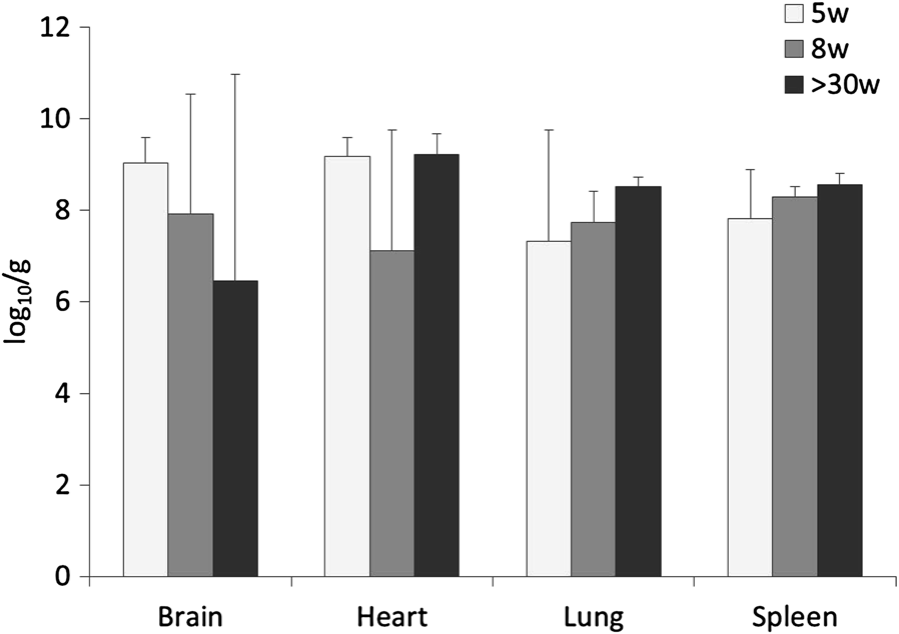
**Virus detection in tissues of broilers inoculated with high dose of A/turkey/Minnesota/12582/2015 (H5N2) virus.** Virus detection by qRRT-PCR. The limit of detection for Tk/MN/15 virus was 3.0 log_10_ EID_50_/g; therefore, qRRT-PCR negative samples were given a numeric value of 2.9 log_10_ EID_50_/g. Virus titers in each type of tissue were statistically similar among the three age groups (Kruskal–Wallis test, *p* > 0.7). Intranasally inoculated 5-week old broilers (5w), 8-week old broilers (8w), >30-week-old broiler breeders (>30w).

Brain, spleen, heart, and lung were collected for viral RNA quantification by qRRT-PCR from two necropsied birds per age group challenged with the high dose of Tk/MN/15 virus. Mean virus titers per tissue were calculated using the two birds from each age group necropsied at 3 dpc. High virus loads were detected in all the tissues and in all necropsied birds, ranging from 6.4 log_10_ EID_50_/g of brain (>30w) to 9.2 log_10_ EID_50_/g of heart (5 and >30w) (Figure 4). Virus titers in each type of tissue were statistically not different among the three age groups (Kruskal–Wallis test, *p* > 0.7).

## Discussion

The H5N2 HPAI virus outbreaks in the Midwest during 2015 affected commercial turkey and layer chicken farms, but not broiler farms [5] despite close proximity of the three types of production systems [32]. To determine if broilers had any genetic resistance to HPAI virus infection and subsequent reduction in contact transmission, we investigated the ability of the Midwestern H5N2 clade 2.3.4.4 Tk/MN/15 virus to produce infection and be transmitted by direct contact among commercial slaughter-age broilers and adult broiler breeders under the stress of laying.

Previously, the experimental BID_50_ of 4.7 log_10_ EID_50_ was determined to be the upper cut-off for sufficient poultry adaptation for sustained transmission and spread of viruses between two or more premises in the field [41]. In the current infectivity and transmission studies, 100% of the birds inoculated with 6.0 log_10_ EID_50_ became infected and died which resulted in an estimated BID_50_ and BLD_50_ for Tk/MN/15 virus of 5.0 log_10_ EID_50_ in all age groups. This concurs with a previous study with 3 and 6w commercial broilers that experienced 100% mortality following inoculation with 6.5 log_10_ EID_50_ of this same virus (Dr Kapczynski, personal communication, 29 July 2016). In our study, the BID_50_ obtained is similar to the upper cut-off value, suggesting inadequate or sub-optimal adaption for sustained transmission in broilers. The MDT was statistically not different among the three age groups, being 4.8 dpc for 5-week-old broilers, and 3.2 dpc for 8-week-old broilers and the >30-week-old broiler breeders. Collectively, the BID_50_ and BLD_50_ results suggest that differences in age are not a determining factor in the susceptibility of broilers for Tk/MN/15 virus, and that this virus lacks optimal adaptation to broilers and/or broilers are mildly genetically resistant to infection by this virus.

Virus shedding was in line with mortality results. Broilers of all ages inoculated with 6.0 log_10_ EID_50_ of Tk/MN/15 virus shed high levels of virus prior to death resulting in transmission to contact exposed birds. The lack of significantly different amounts of virus shed further confirmed the absence of age-related differences in susceptibility. Broilers of all ages inoculated with the low and medium doses of Tk/MN/15 virus did not become infected based on lack of mortality, virus shedding, and seroconversion. Collectively, our findings concur with DeJesus et al. [8] that observed virus shedding from SPF White Leghorn chickens inoculated with a high dose of this same virus and transmission to contact birds. The overall virus shedding titers in broilers at all ages tested suggests a high potential of this virus to transmit within the broiler host population, yet high virus loads would be required to achieve the infectious dose upon initial exposure. However, field conditions with associated secondary infections or immunosuppression might lower such minimal exposure dose, thus emphasizing the importance of good biosecurity and management practices to explain the absence of affected broiler farms in 2015. The lack of age-related effects on susceptibility to Tk/MN/15 virus was also supported by the pathogenesis data; broilers infected with the highest dose of virus showed histopathological findings that were similar among the three age groups, which were consistent with typical Gs/GD lineage H5N1 HPAI viruses [14, 37, 40]. When the three ages of broilers were compared using systemic replication tested by qRRT-PCR, levels of virus found in each tissue were not significantly different.

Recently, 4-week-old turkeys experimentally challenged with Tk/MN/15 virus had the same BID_50_ and BLD_50_ of 5.0 log_10_ EID_50_ as broilers, but a longer MDT of 5.9 dpc [9]. In contrast, a recent study by DeJesus et al. [8] using the same Tk/MN/15 virus had a BID_50_ and BLD_50_ of 3.6 log_10_ EID_50_ and MDT of 2.0 dpc in 4-week-old specific pathogen free (SPF) White Leghorn chickens. All the studies combined suggest that the broiler breed is less susceptible to Midwestern H5N2 poultry viruses than the layer breed, but similarly susceptible as turkeys. Interestingly, 80 and 90% of the layer and turkey farms, respectively, affected during the Midwestern outbreaks were premises with birds over 9 weeks old, and no premises with birds younger than 4 weeks old were affected [42]. These data suggest a potential age-related susceptibility factor in layer hens and turkeys that calls for further experimental confirmation. In any case, a mild genetic resistance to infection might explain only partially why broiler premises were not affected during the 2015 outbreak. The type of production system could have played a greater role than initially expected; the potential chance of virus introduction into broiler premises is likely much lower than in layer premises because broilers are shorter-lived birds and, therefore, faster bird turnover rate occurs with fewer times for workers, equipment, and supplies to enter the premises. This hypothesis might also explain the lack of infected layer chicken and turkey premises holding <4-week-old birds during the 2015 outbreak. In addition, better biosecurity practices on broiler farms than in layer and turkey farms, many of which are directly dependent on the production system, may have prevented exposure on many broiler farms, likely contributing to the absence of outbreaks on broiler premises. While there were broilers at different stages of production within the control and surveillance zones, based upon permits it appeared that some broiler producers did not restock (Dr Mia Torchetti, personal communication). Similarly, in 1996–1998 an H7N2 LPAI virus in Pennsylvania affected layer flocks but not broiler flocks, despite close proximity of infected premises [26, 43]. Also, in the 2014 H5N2 clade 2.3.4.4 HPAI outbreak in British Columbia, Canada, all commercial poultry farms with the exception of chicken broiler farms were affected [44].

In conclusion, the present study indicates that age is not a determining factor in susceptibility of broilers to H5N2 clade 2.3.4.4 HPAI virus. The epidemiology of infected premises of the H5N2 outbreak in the Midwest in 2015 also appears consistent with the data presented in this study. Based on BLD_50_, the Tk/MN/15 virus is slightly less adapted to broilers than to layers, which may partly explain why layer chickens and not broiler chickens were affected during the outbreak. However, the high pathogenicity of the virus and the readiness of replication and transmission in broilers exposed to high doses of Tk/MN/15 virus indicate that good biosecurity practices and disease control measures likely contributed to the prevention of HPAI virus being introduced and spread in broiler premises.

## Abbreviations

5w: 5-week-old broilers
8w: 8-week-old broilers
>30w: >30-week-old broiler breeders
AI: avian influenza
Gs/GD: A/goose/Guangdong/1/1996
H5N2 Tk/MN/15: A/turkey/Minnesota/12582/2015
BHI: brain heart infusion
5w-c: contact 5-week old broilers
8w-c: contact 8-week old broilers
>30w-c: contact >30-week-old broiler breeders
dpc: day post-challenge
dpe: days post-exposure
ECE: embryonating chicken eggs
HA: hemagglutinin
HI: hemagglutinin inhibition
HPAI: high pathogenicity avian influenza
LPAI: low pathogenic avian influenza
MHC: major histocompatibility complex
BID_50_: mean bird infectious dose
BLD_50_: mean bird lethal dose
MDT: mean death time
EID_50_: mean egg infectious dose
na: not applicable
NSL: no significant lesions
qRRT-PCR: quantitative real-time RT-PCR
SPF: specific pathogen free
